# GFP fluorescence peak fraction analysis based nanothermometer for the assessment of exothermal mitochondria activity in live cells

**DOI:** 10.1038/s41598-019-44023-7

**Published:** 2019-05-17

**Authors:** Oleksandr A. Savchuk, Oscar F. Silvestre, Ricardo M. R. Adão, Jana B. Nieder

**Affiliations:** Department of Nanophotonics, Ultrafast Bio- and Nanophotonics group, INL - International Iberian Nanotechnology Laboratory, Av. Mestre José Veiga s/n, 4715-330 Braga, Portugal

**Keywords:** Molecular imaging, Biosensors

## Abstract

Nanothermometry methods with intracellular sensitivities have the potential to make important contributions to fundamental cell biology and medical fields, as temperature is a relevant physical parameter for molecular reactions to occur inside the cells and changes of local temperature are well identified therapeutic strategies. Here we show how the GFP can be used to assess temperature-based on a novel fluorescence peak fraction method. Further, we use standard GFP transfection reagents to assess temperature intracellularly in HeLa cells expressing GFP in the mitochondria. High thermal resolution and sensitivity of around 0.26% °C^−1^ and 2.5% °C^−1^, were achieved for wt-GFP in solution and emGFP-Mito within the cell, respectively. We demonstrate that the GFP-based nanothermometer is suited to directly follow the temperature changes induced by a chemical uncoupler reagent that acts on the mitochondria. The spatial resolution allows distinguishing local heating variations within the different cellular compartments. Our discovery may lead to establishing intracellular nanothermometry as a standard method applicable to the wide range of live cells able to express GFP.

## Introduction

Cells are the basic units of life that can be used to understand various physical and biological processes in the body. Every form of cell activity, like cell division, gene expression, enzyme reaction, metabolism and pathological states are marked by temperature changes. Thus, the knowledge on the temperature of the cells can provide additional information about their function and behavior^1^. In fact, it has been demonstrated that luminescence thermometry provides the prospect of early-stage cancer diagnosis as demonstrated in pre-clinical *in vivo* studies^2^. Moreover, intracellular temperature measurements can be used as a control during hyperthermia treatment, in order to prevent overheating of healthy tissues^3^.

In general, all the intracellular temperature sensors reported so far can be divided in two categories, invasive (micro-thermocoulple) and noninvasive (infrared and luminescence thermometry)^4–7^. Recently, Yang and co-workers were able to perform local temperature measurements close to adherent human hepatoblastoma cells, with a minimum variation of 60 mK, using a micro-thermocouple^8^. Such measurements were continuously recorded for up to 57 hours in an incubator at stabilized temperature. Despite the very high thermal sensitivity of this sensor, it requires a very complicated fabrication of the micro-thermocouple. Since these kinds of thermal probes require physical contact with the system under study, the coupling and inefficiency of the heat transfer from the system to the probe can lead to errors in the temperature measurements. Also, such techniques are limited to surface temperature-measurements.

Alternatively, infrared thermometry provides a non-invasive and non-destructive way to measure temperature. Paulik *et.al*. have achieved a very high thermal resolution of 0.002 °C using infrared thermometry in thermogenesis studies of isolated cells after exposing them to the mitochondrial uncoupler, carbonyl cyanide-4-(trifluoromethoxy) phenylhydrazone (FCCP), or transforming the cells with the uncoupling protein-2^9^. However, this method has some drawbacks such as low spatial resolution and the limitation to surface measurements only. Furthermore, prior knowledge of the material emissivity is required for accurate measurements^4–7^.

Luminescence nanothermometry emerged as a versatile tool for different kinds of applications including intracellular temperature measurements^6^, controlled photothermal and magneto-thermal therapy^3,10^, thermal conductivity measurements^11^, investigating muscle efficiency^12^, microscopic heat production in living organisms^13^ and even for investigating the Brownian motion in ballistic regime^14^. Among the various types of temperature-dependent luminescence variables, the ratiometric technique is often chosen due to its robustness against photobleaching and compared to e.g. fluorescence lifetime-based techniques it requires less specialized excitation and detection equipments. Advantages of ratiometric methods include the use of relatively accessible measurement equipment (common fluorescence microscope) and high temporal resolution (better than 1 s)^15^. Oppositely to other techniques, it does not require the use of expensive pulsed excitation sources, as in the case of fluorescence lifetime temperature-dependent measurements, and it is not limited to certain materials as in the case of Fluorescence Polarization Anisotropy (FPA). Ratiometric intracellular temperature-sensing techniques have mostly been reported using so-called luminescent molecular thermometers, typically with nanoscale probe-sizes and strongly temperature-dependent luminescent properties^15,16^. They include: small organic molecules^17–19^, quantum dots^20–26^, carbon dots^27,28^, upconversion nanoparticles^29^ and polymers^30–34^. However, nearly all of them require complicated synthesis procedures, and most of them are not commercially available.

Green Fluorescent Protein (GFP) has proven to be a remarkable protein for many biological and medical applications^35^. Not surprisingly, in 2008 O. Shimomura^36^, M. Chalfie^37^ and R. Y. Tsien^38^ were awarded the Nobel Prize in chemistry for their discovery and development of GFP as a versatile genetic tool. Based on the different state of the chromophore, there are seven classes of GFP variants: (i) wild-type mixture of neutral phenol and anionic phenolate; (ii) phenolate anion; (iii) neutral phenol; (iv) phenolate anion with stacked π–electron system; (v) indole; (vi) imidazole; (vii) phenyl. The main structural features of the GFP mutants are very well conserved, although they have quite distinct emission and absorption properties ranging from UV to red region of electromagnetic spectrum^38^. Recently, GFP emerged as a promising material for luminescence nanothermometry, due to its remarkable optical properties. However, the implementations reported so far, either require specialized microscopy equipment, e.g. with FPA implementation^39,40^ or advanced molecular biology techniques for gene modification^41,42^. One of the first studies on the usage of GFP as luminescence nanothermometer was reported by Donner and co-workers^40^. They have shown that intracellular temperature measurements can be performed using the FPA of GFP, which is decreasing linearly within biological temperature, in a range of 20–60 °C. They were able to obtain intracellular thermal images of HeLa and U-87 cells and even *in vivo* in *Caenorhabditis Elegans*, where GFP was expressed^39,40^.

One general motivation for the development of intracellular nanothermometers is the potential to study thermogenesis, a fundamental process in cell biology. A theoretical study estimated that intracellular temperature rise by endogenous thermogenesis is not able to exceed 10^−5^ K^43^. However, it has been demonstrated in various chemically-induced thermogenesis in live cells, that changes of >1 K can be observed^19,44,45^. These works revealed several errors in the theoretical model and furthermore, underpin the importance for performing nanothermometry at the ‘heat spot’. Remarkable results were shown by Kiyonaka *et.al*. who developed a genetically-encoded fusion protein that combines GFP with a thermosensitive coiled-coil protein TlpA, into the fusion protein tsGFP^41^. This enabled the visualization of thermogenesis in discrete organelles in living cells. The thermal reading was performed by means of temperature-dependent difference in the 400 and 480 nm excitation peaks of tsGFP. Recently, Nakano *et.al*. developed a genetically encoded Förster Resonance Energy Transfer (FRET)-based thermosensitive hybrid that combines two variants of GFP, which possesses distinct temperature sensitivities^42^. They demonstrated a temperature increase of 6–9 °C induced by FCCP in the mitochondria matrix of HeLa cells. Also they show differences of 2.9 ± 0.3 °C in the temperature across individual cells, between cytosol and nucleus areas. Although high intracellular thermal sensitivity can be achieved using such method, it requires time consuming gene encoding. Also, small emission spectral separations of fluorescent proteins can lead to FRET that can compromise the performance of the nanothermomer.

Here, we report luminescent nanothermometry based on analyzing distinct Peak Fractions (PF) of the fluorescence emission of GFP protein in solution or within cells, using versatile and easy to handle commercially available GFP transfection reagents. Such PF measurements take advantage of the ratiometric technique being independent on sample concentration and excitation power variations. Another advantage over engineered nanostructured materials or molecules being inserted into the cells, is that the proposed intracellularly expressed proteins do not require any additional biocompatibility screenings. Our genetically encoded nanothermometer technique is based on a PF parameter of the fluorescence emission spectrum of GFP. The first proof of concept application of our genetically encoded nanothermometer technique is performed to determine chemically-induced heat production in the mitochondria regions of the HeLa cells.

## Results

### wt-GFP as a nanothermometer

**Figure 1:**
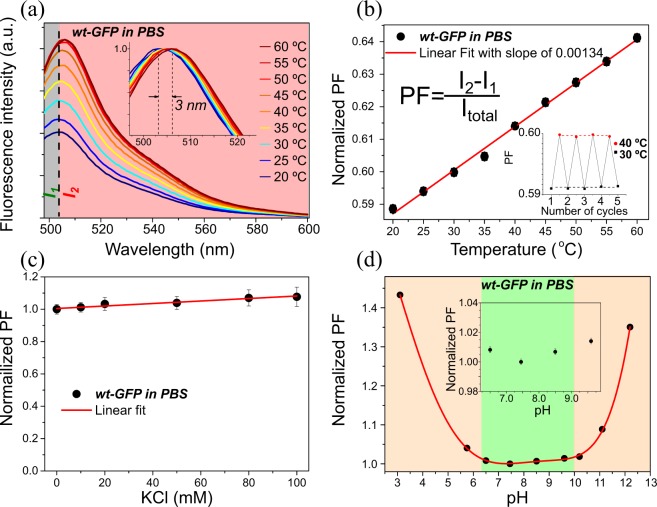
Characterization of the molecular nanothermosensor wt-GFP. (**a**) Temperature-dependent emission spectra of GFP excited at 488 nm. Inset showing red shift of 3 nm at elevated temperature; (**b**) PF of fluorescence emission spectra of wt-GFP in PBS upon temperature increase with the inset of reproducibility during five cycles; (**c**) Ionic strength and (**d**) pH dependence determined at ambient temperature of 22 °C and with the PF parameter (PF = I_2_ − I_1_/I_total_) normalized to the 0 mM of KCl and pH 7.5, respectively. A red line connecting the data points was added, the green area highlights the PF-stable pH range and an inset shows a zoom into the physiological relevant range.

We analyze the temperature-dependent fluorescence emission spectra of wt-GFP excited at 488 nm shown in Fig. 1a. It can be observed that the fluorescence emission intensity increases at elevated temperatures. In order to analyze this behavior, excitation spectra of wt-GFP were obtained using an emission at 504 nm (Fig. S1). As the temperature increases from 25 °C to 60 °C, the neutral band at 395 nm decreases in intensity, while the anionic band at 475 nm slightly increases, resulting in higher fluorescence emission at elevated temperatures. The mechanisms of fluorescence emission of GFP are well known and can be explained as follows^46^: the ground state of GFP can exist in both anionic (B) and neutral forms (A). The anionic form absorbs at about 475 nm, whereas the neutral protonated form absorbs at about 395 nm. The absorbance band at 395 nm is 3 times higher than the minor peak at 475 nm. Excitation of either neutral or anionic form results in green fluorescence at 504 nm from the anionic state.

Although, in wt-GFP the variation of the fluorescence emission intensity with temperature is quite prominent (see Fig. 1a) and for the variant EGFP has been used to determine intracellular temperature^47^, this fluorescence parameter is prone to various artifacts due to photobleaching, inhomogeneous concentration, excitation power instability, ionic strength and pH variations in the surrounding medium. Therefore, a perfect luminescence nanothermometer would have a temperature-dependent parameter insensitive to those factors. Besides the increase of fluorescence emission intensity, a red shift of the emission spectra of about 3 nm can be observed and is highlighted in the inset of Fig. 1a. This spectroscopic feature was reported in the Aequorea GFP and attributed to a dipolar moment variation, resulting from a charge density transfer occurring between the close neighbors oxygen atoms^48^. Dipolar change reorganizes molecules around the electronic excited state decreasing its energy leading to observed red shift of emission maximum^49^.

Thus, we define a PF parameter as:

1 $${\rm{PF}}=({{\rm{I}}}_{2}-{{\rm{I}}}_{1})/{{\rm{I}}}_{{\rm{total}}},$$

where I_1_ and I_2_ are the integrated intensities between I_1_: 495–504, and I_2_: 505–600 nm, respectively and I_total_ is the total fluorescence emission intensity of wt-GFP. The integrated intensity I_1_ represents the area before the fluorescence emission peak maximum and I_2_ the area after the peak maximum. In this way, we achieve a method to quantify the temperature-induced fluorescence emission peak shift towards red region of electromagnetic spectrum. The so defined PF is independent of the total intensity of the sample, as the amount of peak shift is normalized to the total intensity. Thus, it is independent of sample concentration and power fluctuations of the excitation source.

We discover that the PF follows a linear increase with temperature within the biologically relevant temperature range from 20 to 60 °C (Fig. 1b). The inset in the Fig. 1b represents the good repeatability of the PF measurements during four sequentially performed temperature cycles between 30 and 40 °C. Moreover, we find that the PF is almost not affected by environmental conditions. The performance of the wt-GFP nanothermometer was tested under varied conditions of ionic strength (Fig. 1c) and pH (Fig. 1d). Potassium chloride was used as an ionic source to reproduce intracellular conditions. The results shown in Fig. 1c indicate negligible dependence of the PF on the ionic strength. The pH of wt-GFP PBS buffer was modified by adding NaOH or HCl. As it can be seen from Fig. 1d, the PF is almost independent on the buffer pH in the wide range from 6.5 to 10.5 (highlighted in green). Acidic solutions with a pH lower than 6.5 and basic solutions with pH higher than 10.5 lead to strong distortions of the fluorescence emission spectrum of wt-GFP, causing strongly altered PF values. To provide a closer look into the pH dependency of the PF parameter around the physiological relevant range, we provide an inset for the pH range from 6.5 to 9.5, for which the PF variation only amounts to about 1%.

The thermal resolution of a luminescent nanothermometer can be calculated according to:

2 $${\rm{\delta }}T=(\partial {\rm{T}}/\partial {\rm{PF}}){\rm{\delta }}\mathrm{PF},$$

where ∂T/∂PF represents the inverse slope of the PF parameter as a function of temperature, and δPF is the standard deviation of the PF. For wt-GFP, the thermal resolution is found to be around 0.75 °C. In fact, the first intracellular temperature measurements were performed using a similar technique, where the 6-dodecanoyl-2-dimethylamino-napthalene (laurdan) undergoes a gel-to-liquid crystalline phase-transition as temperature increases^50^.

While the linear temperature dependence and sensitivity of wt-GFP is promising for nanothermometry applications *in situ* (in PBS), the poor ability of wt-GFP to mature in warm environment, as well as UV excitation, has led to some restrictions in cell biology studies. GFP variants, like emGFP or EGFP, with phenolate anions in the chromophore have appeared to overcome such problems. In particular, emGFP displays improved photostability and brightness, higher expression level at 37 °C, suitability for cell biology and simple excitation and emission spectra have been achieved. The excitation peak observed at 395 nm for wt-GFP, is suppressed due to neutral phenol, and the 475 nm peak is enhanced five-to-six fold and red shifted to 490 nm^51^. In this context, baculovirus technology has attracted a lot of attention due to its efficient protein production in insects and mammalian cells, with possibility of gene-delivery vectors applications^52,53^. It provides accurate and efficient targeting to specific cellular structures with minimal toxicity and technical simplicity. In our work we use commercially available CellLight BacMam 2.0 expressing emGFP fused to the leader sequence of E1 alpha pyruvate dehydrogenase multienzyme complex, located in the mitochondrial matrix.

### Intracellular GFP based nanothermometry at the mitochondria

It has been reported that heat activation induces apoptosis starting from mitochondria matrix of the cell^54^. Thus, it is important to monitor intracellular temperature directly at mitochondria. In order to study the fluorescence emission spectrum arising from expressed emGFP inside the human cervical cancer cells (HeLa) at a mitochondria region, commercially available CellLight BacMam 2.0 Mitochondria-emGFP (emGFP-Mito) was used. Colocalization analysis with Mito Hunt Red was performed in order to ensure the mitochondria localization of emGFP-Mito (see Fig. S2). Temperature dependence of the fluorescence emission intensity arising from emGFP-Mito was evaluated with the spectral detection mode available in the Zeiss LSM 780 confocal microscope. Figure S3 in Supplementary Information shows the fluorescence emission spectrum of expressed emGFP-Mito inside the HeLa cells that was obtained by integrating the full image recorded at stabilized temperatures of 20 and 35 °C. In this case, the fluorescence emission of emGFP-Mito displays a maximum peak located at 509 nm for 20 °C, which significantly drops in intensity with increasing temperature, while the peak experiences a red shift to 512 nm at 35 °C.

Temperature dependence of the fluorescence intensity of the emGFP-Mito is inverse compared to wt-GFP. In the case of emGFP-Mito increased temperature induces reduction of the hydrogen-bond network resulting in a decrease of fluorescence intensity as has been observed elsewhere^48^. The fluorescence intensity images of HeLa cells expressed with emGFP-Mito at 23–39 °C temperature is shown in Fig. S4. Mean fluorescence intensity value calculated from the Fig. S4 as a function of temperature is presented in Fig. S5a. Moreover, the temperature-induced fluorescence peak shift of emGFP-Mito is more sensitive towards temperature change compared with wt-GFP. While for emGFP-Mito the shift of 3 nm occurs in a smaller temperature interval from 20 °C to 35 °C, in case of wt-GFP a 3 nm shift occurs for a temperature change from 20 °C to 60 °C.

**Figure 2:**
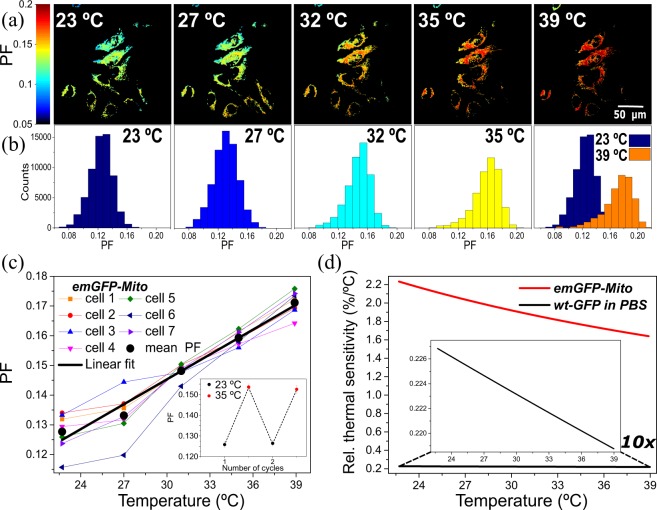
Characterization of the intracellular molecular nanothermosensor emGFP-Mito. (**a**) PF images of the HeLa cells containing emGFP-Mito at different temperatures; (**b**) Histograms of the PF parameters found in the areas with emGFP-Mito signal; (**c**) Temperature dependence of the PF parameter of emGFP-Mito transfected in HeLa cells for individual cells and mean behaviour. An inset shows results of a repeatability study; (**d**) Relative thermal sensitivity calculated for emGFP-Mito at 23–39 °C temperature range, compared with wt-GFP in PBS at the same temperature range.

To demonstrate the temperature sensing capability using the PF parameter of the emGFP-Mito fluorescence, first we perform intracellular calibration experiments with emGFP-Mito expressed in HeLa cells. In the case of emGFP-Mito, the intensities are obtained in the emission ranges of I_1_: 495–509 and I_2_: 510–600 nm, respectively. We use acousto-optical tunable filters in-built in the Zeiss LSM 780 confocal microscope to project the selected spectral ranges onto two distinct detection channels of a multichannel PMT detector. The temperature-dependent PF images (Fig. 2a) are shown together with the associated histograms of the PF values according to Eq. 1 found in the individual pixels of the images (Fig. 2b). Associated fluorescence intensity per channel and differential interference contrast (DIC) image are shown in Fig. S4. The PF value is slightly different among the cells, which may be related to the different metabolic activities of an individual cells. The emGFP-Mito thermosensor calibration curve (Fig. 2c, thick black curve) was determined as the mean value of the PF over areas of individual cells (Fig. 2c, colored thin curves). An inset shows results of a repeatability study. The PF images and representative histograms during cooling/heating cycle at 23 and 35 °C, respectively, is shown in Fig. S6. As in the case of wt-GFP in PBS, a linear increase of the PF upon temperature rise can be seen for emGFP-Mito within HeLa cells. As live cell experiments above 40 °C are difficult to perform experimentally due to limited set temperature ranges of standard incubators and additionally the risk of inducing cell death, we assume in analogy to wt-GFP a continuation of the linear behavior up to 60 °C. The thermal resolution of emGFP-Mito is found to be around 0.26 °C according to Eq. 2.

A quantitative comparison between different luminescent nanothermometers is possible by comparing the relative thermal sensitivity S_rel_^4,7^. We calculated S_rel_ for both sensors using

3 $${{\rm{S}}}_{{\rm{rel}}}=(\partial {\rm{PF}}/\partial {\rm{T}})/{\rm{PF}}{\rm{.}}$$

The relative thermal sensitivities for wt-GFP and emGFP-Mito are presented in Fig. 2d. For wt-GFP the maximum was found to be 0.23% °C^−1^, which corresponds to the same order of magnitude that was found for GFP using FPA technique^40^. The relative thermal sensitivity of the emGFP-Mito was found to be around ten times higher than for wt-GFP in PBS with maximum at 2.2% °C^−1^ (Fig. 2d). Table 1 presents a comparison between existing GFP-based nanothermometers tested *in vitro*. The major difference lies in the thermometric parameter response upon temperature rise. In this work, the PF parameter is increasing linearly at elevated temperature which leads to a negative slope of relative thermal sensitivity. Other GFP-based nanothermometers based on the ratiometric, fluorescence intensity or FPA approach decrease linearly with temperature increase, which results in a positive slope of the relative thermal sensitivity. Interestingly, for the PF-based technique of emGFP-Mito the sensitivity reaches the maximum sensitivity at lower temperatures which might be of benefit for experiments conducted at ambient temperatures, while at optimal live cell experimental conditions around 37 °C the emGFP-Mito performs at reasonable sensitivity. While the intensity-based approach reaches higher sensitivity values with maximum 4.4% °C^−1^ at 39 °C (see Fig. S5b), the technique is not chosen to be appropriate for cell imaging studies that require dynamic observations, with non-negligible photobleaching between subsequent scans. The relative thermal sensitivity of the PF emGFP-Mito is among the highest ever reported. Besides the em-GFP-Mito intensity-based approach, only genetically-encoded fusion of GFP with coiled-coil protein TlpA expressed in the plasma membrane (tsGFP1-F) and endoplasmic reticulum (tsGFP1-ER) of HeLa cells have higher relative thermal sensitivity. However, the main drawbacks of this technique are the need of two excitation sources to be used simultaneously and a complicated synthesis procedure. Also, the same type of fusion system expressed in the mitochondria, tsGFP-mito, has lower relative thermal sensitivity compared to emGFP-Mito. As it can be seen in Table 1, emGFP-Mito using the PF-based sensing parameter shows superior performance compared to the fluorescence intensity-based EGFP system^47^ and the ratiometric FRET-based thermosensitive hybrid with two GFP variants gTEMP expressed at the mitochondria^42^. Furthermore, our system has one order of magnitude higher thermal sensitivity and two times higher thermal resolution than FPA-based luminescent nanothermometry technique achieved by Donner and co-workers^40^. The higher relative thermal sensitivity of emGFP-Mito can be attributed to parameters such as the improved brightness of this GFP variant, more sensitive temperature-induced peak shift, the distinct media and/or the detection system used in the experiments. Thus, it is crucial to obtain a calibration curve for each experimental detection system used in our studies.

**Table 1:** Comparison of relative thermal sensitivity of different GFP-based luminescent nanothermometers tested *in vitro*.

Material	Sensing parameter	T range, °C	T resolution, °C	S_rel_^max^(T), % °C^−1^	*In vitro* model	Localization	Ref.
**emGFP-Mito**	**PF**	**23–39**	**0.26**	**2.2 (23)**	**HeLa**	**Mitochondria**	**This work**
tsGFP1-ER	Ratio	40–47^ǂ^	—	2.82 (47)*	HeLa	Endoplasmic reticulum	^41^
tsGFP1-F	Ratio	40–45^ǂ^	—	2.28 (45)*	HeLa	Membrane	^41^
tsGFP1-mito	Ratio	35–45^ǂ^	—	1.54 (45)*	HeLa	Mitochondria	^41^
EGFP	Intensity	20–60	—	1.23 (60)*	Escherichia coli	—	^47^
gTEMP	Ratio	34–40	0.4	1.15 (34)*	HeLa	Mitochondria	^42^
GFP	FPA	24–40	0.4	0.5 (40)*	HeLa and U87 MG	Cytoplasm and nucleus	^40^

*Estimated from the calibration curve using Eq. 3.

^ǂ^linear region of the analyzed temperature range.

### Intracellular heat production at the mitochondria

For the application and testing of luminescent nanothermometers inside of cells, several controllable intracellular heating processes have been proposed^6,55^. Basically, they can be divided into two categories: heat production due to chemical stimuli, using mitochondria uncouplers, which accelerate thermogenesis of the cell, and heat production induced by photo-thermal e.g. plasmonic materials located close or inside the studied cells. We choose to promote the intracellular temperature by a chemical stimuli, since in this case our nanothermometer is in direct vicinity to the mitochondria source of heat. Some work indicates there might be a pH change associated to mitochondria uncoupler reagents such as FCCP^56^, which so far has been neglected in works on nanothermometry. While the effect of pH change on the PF parameter of emGFP-Mito inside of live cells is difficult to assess, we perform experiments on fixed cells instead (See Fig. S7). This allows for equilibration with the pH and KCl ionic strength adjusted PBS. Interestingly, we find neglectable dependence of both parameters on the PF of emGFP-Mito.

**Figure 3:**
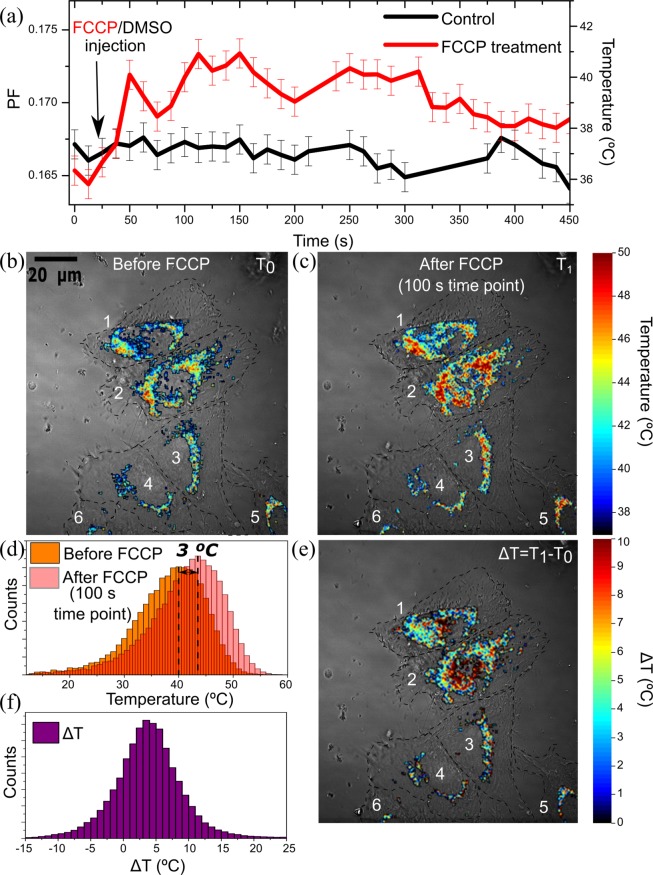
Heat production by chemical stimuli. (**a**) Temporal response of the heat production by chemical stimuli of the HeLa cells transfected with emGFP-Mito (the lines represent mean values of all the cells); (**b**) Thermal image of the HeLa cell transfected emGFP-Mito before and (**c**) after FCCP overlapped with DIC images (dashed black lines follow the edge of the cells; (**d**) Histograms of the temperature distribution before and after FCCP treatment; (**e**) Temperature difference image after heat production with FCCP uncoupler; (**f**) Histogram of the temperature difference.

Temperature sensing is demonstrated by performing chemically-induced thermogenesis of the emGFP-Mito transfected HeLa cells, by adding the mitochondria uncouplers FCCP. The results are presented in Fig. 3a and show the evolution of the mean PF value of all the cells (left y axis) and the derived intracellular temperature evolution (right y axis) in mitochondria regions before, during and after addition of FCCP. The measurements were acquired with a 12.5 s time resolution and during an observation period of 7 minutes. The raw intensity images per channel are shown in Fig. S8. We estimate the PF accuracy via the PF standard deviation for 5 consecutive images recorded at stable temperature conditions. With an PF accuracy of about δPF = 0.002, a temperature resolution of δT = 0.5 °C is reached. The control and the FCCP curve are prone to some noise level. Nevertheless a clear response to the FCCP addition can be observed in form of a significant single step where the PF value changes from about 0.167 to 0.172. The derived intracellular temperature is 37 °C before and 40 °C after the step. Subsequently after some fluctuations the temperature stays at elevated levels reaching a maximum of 41 °C after 100 s. This observation indicates that heat is generated immediately after injection of the FCCP uncoupler. After 300 s of FCCP addition, the PF and thus the temperature values decrease. As the availability of nutrients in the intracellular medium (i.e. glucose) is limited, the FCCP-induced reaction may not further be sustained, leading to an effective lowering of the intracellular temperature. The PF parameter shows some fluctuations that relate to a noise level similar to the one observed in the control experiment. On the other hand, the DMSO control experiment does not induce changes of the PF value. Different behaviour among three cells can be related to the cell-cycle, as explained elsewhere^57,58^.

In the Fig. 3b, PF thermal images before and 100 s after the FCCP injection time point are shown. Before FCCP treatment, some parts of the cells have higher temperature than the culture media, which was maintained at 37 °C. A similar behavior was demonstrated recently using thermo-yellow-mito luminescent nanothermometer, in human embryonic kidney (HEK) 293 and primary skin fibroblasts, where this phenomenon was associated to full activation of respiratory chain^59^. Figure 3d shows the histograms of the temperature distribution before and after FCCP treatment. A clear temperature peak shift of 3 °C after FCCP treatment to higher values is observed. For closer examination of the temperature increase inside mitochondria region, the temperature difference between Fig. 3b,c is represented in Fig. 3e. No serious alterations of a mitochondria morphologies that could relate to viscosity changes are noticeable, which indicates that the dynamic changes of the PF parameter are due to intracellular temperature variations. A histogram of the temperature difference is plotted in Fig. 3f, where the maximum abundance occurs at ΔT = 3 °C. Recently, different intracellular temperatures were observed within individual cells, more specifically, with temperature differences between the nucleus and cytoplasm of few Celsius degrees^42,60^. The achieved spatio-thermal sensitivity allows to assess intracellular differences and thereby opens the way to subcellular analysis of temperature gradients, with sub micrometer resolution.

## Discussion

Here, we observe heterogeneity even between different mitochondria parts of the cells. In particular, we observe that mitochondria regions located closer to the nucleus are much warmer. This follows a previous observed trend^57^. There might be a possibility, that there is a direct relation between the mitochondria and the nucleus, where proximity could be important. Mitochondria-to-nucleus communication plays a key biological role, namely, mitochondria biogenesis is controlled by the nucleus, most mitochondria proteins are encoded in the nucleus and many other important signalling metabolic mechanisms depend on the interplay between both organelles. Another possibility might be related with the fact that typically the mitochondria are more densely organized around the nucleus, which may lead to average higher temperatures in this zone. We reproduce the FCCP uncoupler experiments with PF emGFP-Mito detection (see Figs S9 and S10) using a different microscope configuration with a lower magnification of 40x to obtain a larger field of view for the simultaneous detection of a larger, statistically more representative subset of about 20 cells. The determined average temperature after addition of FCCP again increases by several degrees and reaches a temperature difference up to about ΔT = 5 °C after 300 s reaction time. The developed emGFP-Mito-based thermosensing technique has the potential to allow new insights into exothermal biochemical reactions related with metabolic activity of biological cells. In this work we demonstrate the existence of intracellular temperature variations between mitochondria regions, thus highlighting the suitability of emGFP-Mito as a PF-based molecular nanothermometer. Chemically-induced heating of the mitochondria was observed with an average temperature increase of ΔT = 3 °C upon addition of FCCP, which is in good agreement with previously reported values^19,44,45^.

## Conclusion

In summary, we demonstrate the capability of wt-GFP for solution-based and emGFP-Mito for intracellular temperature measurements. The simplicity of adding thermal sensitivity to cells via genetic expression using a commercially available system is established. Other intracellular nanothermosensing requires complicated synthesis and administration of temperature probes and for each cell line the cytotoxicity profile needs to be characterized, while GFP expression induced by baculovirus technology is considered of low toxicity. High relative thermal sensitivity of 0.23% °C^−1^ for PF fluorescence analysis of wt-GFP, and about ten times higher sensitivity of 2.2% °C^−1^ for emGFP-Mito in HeLa cells was achieved with confocal spatial resolution. Thus, not only do we show that simple GFP transfection of cells, combined with a PF calibration, has the potential to significantly facilitate intracellular thermometry, but also we deliver an advanced nanothermometry toolkit to standard cell biology labs, which are often equipped with bioimaging fluorescence microscopy tools to perform intracellular temperature screenings.

We assess the intracellular temperature variation at the mitochondria during treatment with FCCP as a proof-of-concept, which is known to chemically induce heating of the mitochondria. In accordance to previous works, we report an average temperature variation of ∆T = 3 °C upon addition of FCCP^19,44,45^. Additionally, we perform subcellular analysis of temperature gradients with sub-micrometer resolution, and observe heterogeneity between mitochondria located closer or further away from the cell nucleus. We believe that heat induced red shift of the fluorescence emission spectra is common for fluorescent proteins in general. Thus, there may be the possibility to develop a system more suited for subtissue nanothermal assessment, e.g. in the near infrared range. In future work, we propose to assess temperature-dependent fluorescence emission spectra for a representative set of fluorescent proteins.

The new easy and versatile technique of assessing intracellular temperature harbors the potential of creating novel knowledge about biological processes inside the cells, associated to biomolecular reactions at specific organelles, such as mitochondria, which can promote new pathways for diagnosis and therapies of diseases.

## Methods

### Materials

The Penicillin-Streptomycin 100x solution, 0.25% Trypsin-EDTA, and Phosphate-Buffered Saline (PBS) were supplied from Corning Cellgro. HyClone Fetal Clone III serum was obtained from GE Healthcare and Modified Eagle’s Medium (MEM) was purchased from Biowest. Carbonyl cyanide 4-(trifluoromethoxy) phenylhydrazone (FCCP) and wt-GFP solution in PBS was purchased from Sigma. Celllight BacMam 2.0 Mito-GFP was provided by Thermofischer.

HeLa cell line was purchased from ECACC (Cat. No. 93021013), grown under standard culture conditions and seeded inside of two silicone inserts wells on a 35 mm glass-bottom dish (Ibidi) in MEM culture media supplemented with 10% serum, 1% of penicillin-streptomycin, incubated under 5% of CO_2_ at 37 °C. CellLight Mitochondria-GFP BacMam 2.0 was added to the cells in appropriate proportion following the supplier’s protocol and incubated overnight.

### Fluorescence spectroscopy using a Fluorometer

Temperature-dependent fluorescence emission spectra of wt-GFP were measured using a Fluoromax-4 Horiba spectrometer. Wt-GFP solution in PBS with the concentration of 7.4∙10^−7^ M was immersed in the quartz cuvette and placed in the sample holder of the spectrometer. Sample was excited with 488 nm wavelength and the temperature of the sample holder was changed by Peltier-based device with accuracy of ±0.1 °C. Ionic strength dependence experiment was recorded by adding potassium chloride in wt-GFP solution in PBS. The pH dependence of the PF of wt-GFP solution in PBS was measured by preparing two samples, one was used for testing acidic pH by adding HCl and another for bases by adding NaOH.

### Fluorescence spectroscopy using a confocal microscope

Temperature-dependent fluorescence emission spectra of emGFP-Mito in Hela cells were measured using a Zeiss LSM 780 confocal microscope in lambda mode. The microscope is equipped with an incubator that can be used to control the temperature of the sample. For the calibration measurements the temperature was equilibrated for 30 min before each imaging experiment. The temperature reading was performed by means of thermocouple that was directly immersed in the culture media containing the imaging sample with accuracy of ±0.1 °C. Sample was excited with a 488 nm argon laser and signal collected using 63x oil immersion objective. In this system, the fluorescence emission light is separate into its spectral components by using diffractive grating and the detection is given by projecting the entire spectrum on a fixed array of 32 GaAsP detectors. Intensity images of the emGFP-Mito expressed HeLa cells ranging from 496 to 592 nm within a spectral bandwidth of 3 nm at 20 and 35 °C were recorded. Fluorescence emission spectrum was calculated by integration of all pixels (512 × 512) of entire image (100 μm × 100 μm).

### Confocal imaging for PF analysis of emGFP-Mito

The confocal microscope images were taken on Zeiss LSM 780 inverted microscope described above using the 488 nm argon laser. If not mentioned differently a 63x oil immersion objective was used. The fluorescence signal from emGFP-Mito was divided into two channels: I_1_: 495–509 nm (green) and I_2_: 510–600 nm (red) that were recorded simultaneously with 1024*1024 px. For repeatability studies a waiting time of 2 h was used for the cooling step from 35 °C to 23 °C degrees and 40 min waiting time was considered sufficient for the heating step.

Experiments in Fig. S9, were recorded using a 40x oil immersion objective with numerical aperture of 1.2.

### pH and Ionic strength dependence of PF of emGFP-Mito

HeLa cells were transfected with emGFP-Mito using appropriate amount of reagent. After, HeLa cells were washed with PBS and loaded with 4% of paraformaldehyde solution in PBS followed by 20 min of incubation at room temperature. Later, paraformaldehyde solution was removed and cells were washed few times with PBS. Finally, pH of emGFP-Mito was modified by adding NaOH and/or HCl to PBS solution onto fixed HeLa cells. KCl was used as ionic source. Fixed HeLa cells expressed with emGFP-Mito were loaded with 50 and 100 mM of KCl in PBS. Samples were imaged as described under confocal imaging for PF emGFP-Mito analysis.

### Colocalization study of emGFP-Mito with the mitochondria

HeLa cells transfected with emGFP-Mito were incubated with 100 nM of Mito Hunt Red (Setareh Biotech) during 45 min. After, cells were washed with pre-warmed PBS and added 1 mL of culture media.

In the Zeiss 780 confocal microscope Mito Hunt Red was excited with 561 nm diode laser and signal was collected between 595–650 nm.

Colocalization was determined using the 2010 Zen software and Manders Overlap Coefficient analysis.

### Detection of heat production during FCCP treatment

HeLa cells expressed emGFP-Mito cultured inside of two well on a 35 mm glass-bottom dish was placed in the Zeiss LSM 780 confocal microscope equipped with incubator. Temperature was maintained at 37 °C inside the culture media and monitored by thermocouple. First, FCCP was diluted with MEM solution to 200 μM. After, 4 μL of the MEM solution containing FCCP was added to a well containing 80 μL of culture media and HeLa cells expressing emGFP-Mito, diluting FCCP to a final concentration of 10 μM. Identical concentration of DMSO in MEM solution was used for the control experiment. In order to prevent temperature fluctuation while opening the incubator during FCCP treatment, two microfluidics tubes preloaded with 4 μL of FCCP and 4 µL of DMSO solutions were directly placed inside of the culture media, and reaching outside the incubator, where syringes were used to release the reagents. Time series of multichannel fluorescence images from emGFP-Mito expressing HeLa cells were collected every 12.5 s during 450 s. First, control experiment with DMSO solution was recorded followed by the FCCP loading on the same group of cells.

### Data analysis

Images were analyzed using the Zen 2010 imaging processing software provided by Zeiss and MATLAB R2013a. Zen 2010 was used to increase contrast of the images presented in Figs S4 and S8. In order to eliminate background noise signal, a signal threshold of 5 counts per second was subtracted from the images. All the images of the PF values were 2D smoothed using a mean filter of 5 digits. Any data points considered as “not a number” (NaN) after PF calculation were ignored in the averaging. MATLAB R2013a was used to calculate mean values, histograms, and selection of region of interest using an algorithm that allows freehand selection of areas in Figs 2a, 3b,c.

## Supplementary information

Supplementary Information

## Data Availability

All data generated during or analysed during this study are included in this published article (and its Supporting Information files).
