# Supplementary material for: GFP fluorescence peak fraction analysis based nanothermometer for the assessment of exothermal mitochondria activity in live cells
Source: Sci Rep. 2019 May 17;9:7535. doi: 10.1038/s41598-019-44023-7 (PMC6525231; doi:10.1038/s41598-019-44023-7)
Supplement: Supplementary file 1 — Supplementary Information [file 41598_2019_44023_MOESM1_ESM.docx]

**Supplementary information**

GFP fluorescence peak fraction analysis based nanothermometer for the assessment of exothermal mitochondria activity in live cells

Oleksandr A. Savchuk, Oscar F. Silvestre, Ricardo M. R. Adão, Jana B. Nieder*

Department of Nanophotonics, Ultrafast Bio- and Nanophotonics group, INL - International Iberian Nanotechnology Laboratory, Av. Mestre José Veiga s/n, 4715-330 Braga, Portugal


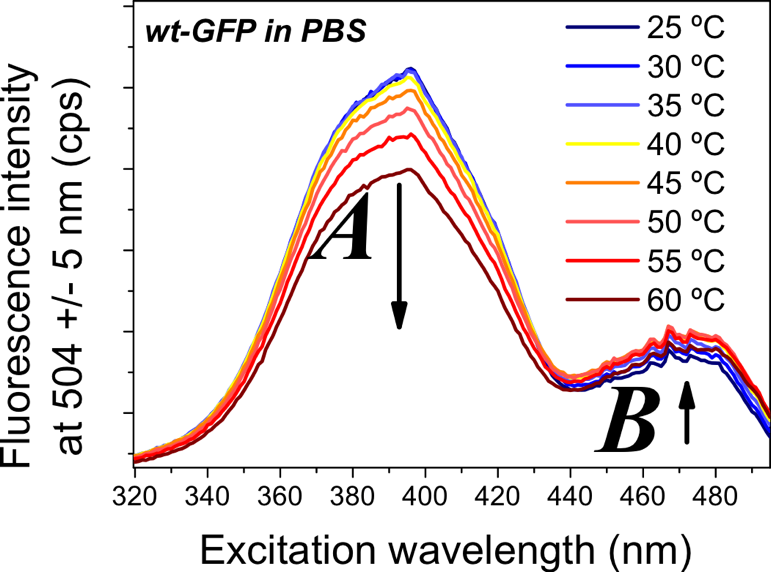


**Figure S1.** Temperature-dependent fluorescence excitation spectra of wt-GFP in PBS between 25 and 60 ºC collected at 504 +/-5 nm. The decrease of neutral (A) and rise of anionic forms (B) are indicated by arrows


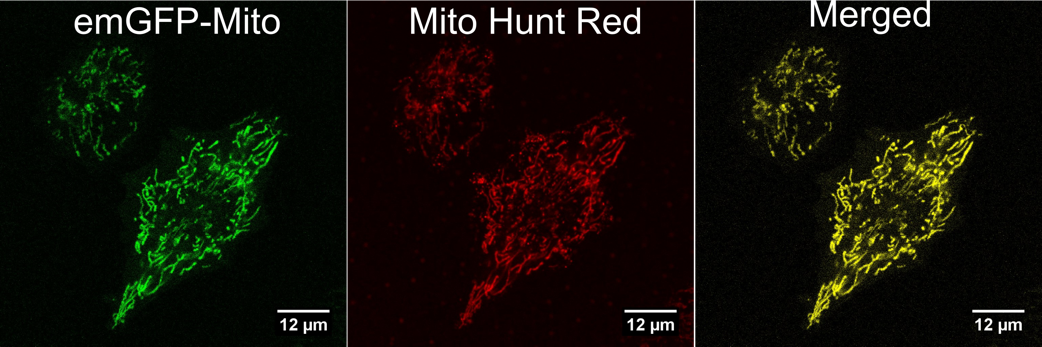


**Figure S2**: Colocalization of emGFP-Mito with the mitochondria using the Mito Hunt Red label to test colocalization. Images show the emGFP-Mito channel (left), the Hunt Mito channel (center) and the merged channels (right) with yellow indicating colocalization of emGFP-Mito and Mito Hunt Red signal. The Manders Overlap Coefficient was determined to be MOC = 0.9.


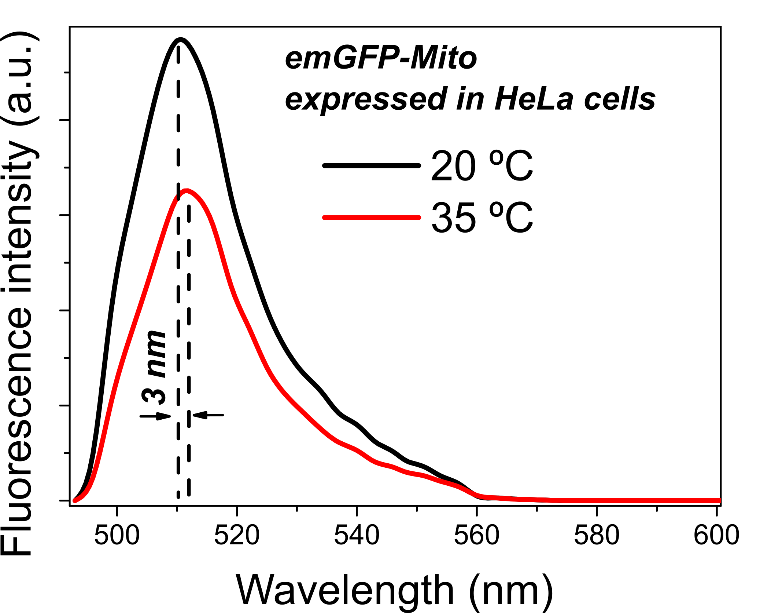


**Figure S3.** Temperature-dependent fluorescence emission spectra of emGFP-Mito excited at 488 nm and measured at 20 and 35 ºC using a Zeiss LSM 780 confocal microscope using lambda mode and averaging over an image area of 100 µm × 100 µm that contains emGFP-Mito expressing live HeLa cell. A red shift of the emission spectra of about 3 nm can be observed.


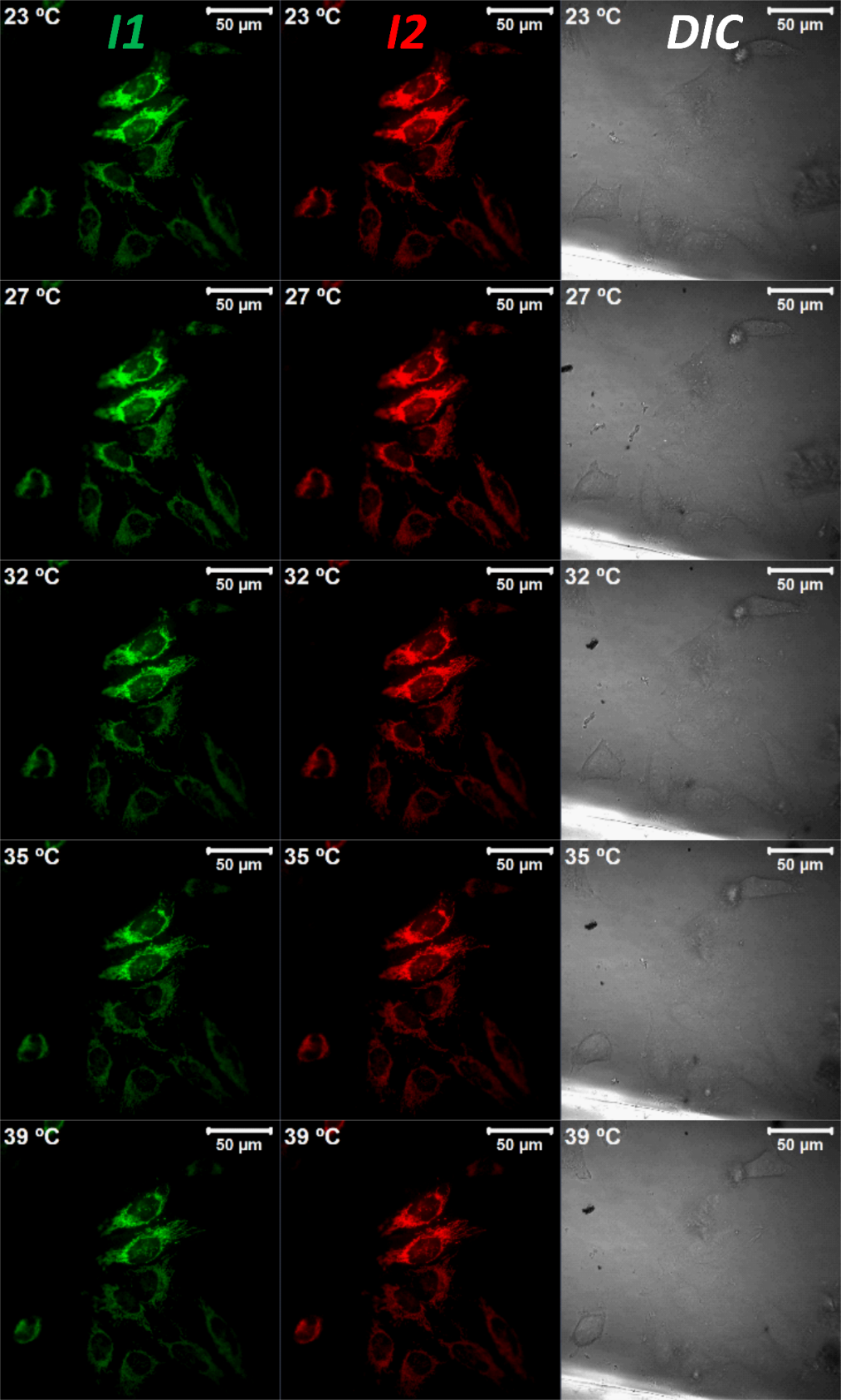


**Figure S4.** Multicolor confocal fluorescence intensity images of emGFP-Mito expressing HeLa cells at 23, 27, 32, 35 and 39 ºC recorded simultaneously in the channel I1: 495 – 509 nm (left), I2: 510 – 600 nm (middle) and in differential interference contrast (DIC) mode (right).


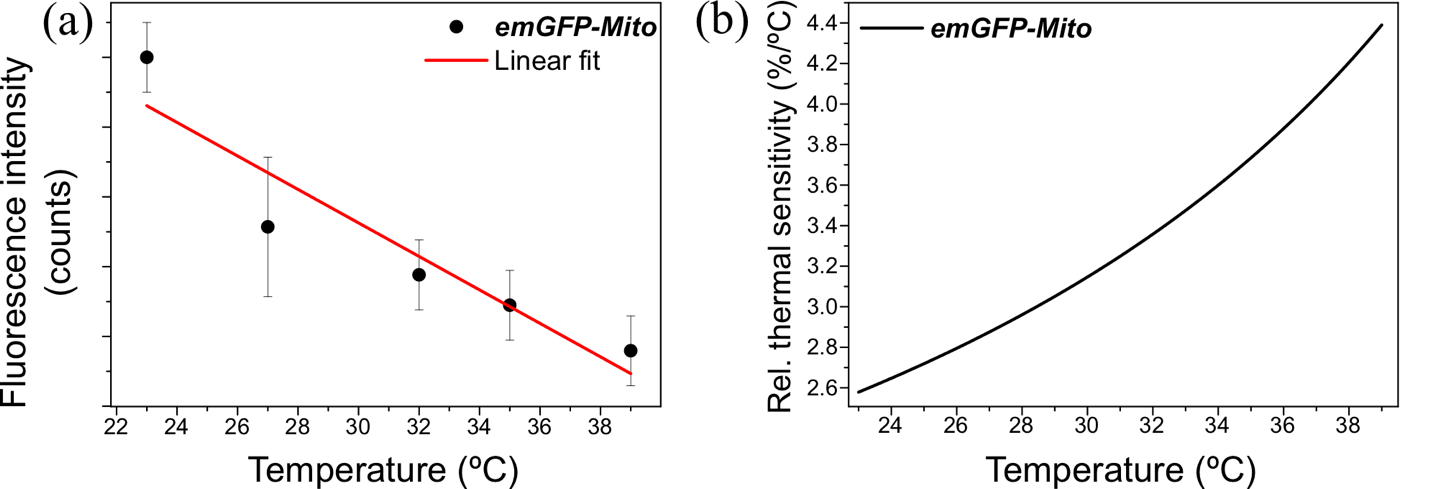


**Figure S5**. (a) Mean fluorescence intensity value of the emGFP-Mito as a function of temperature and (b) Relative thermal sensitivity of emGFP-Mito based on fluorescence intensity parameter


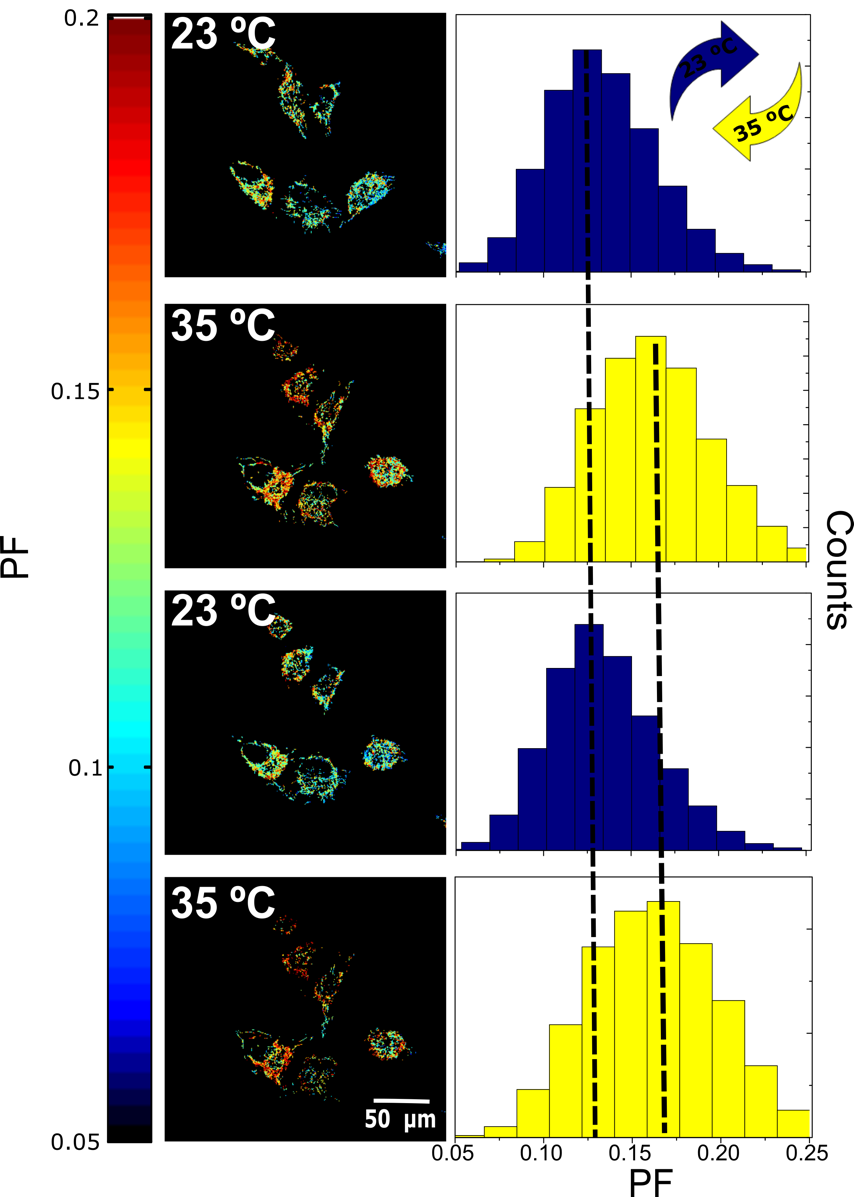


**Figure S6.** Repeatability measurements of PF in emGFP-Mito during heating/cooling to 35 and 23 ºC, respectively.


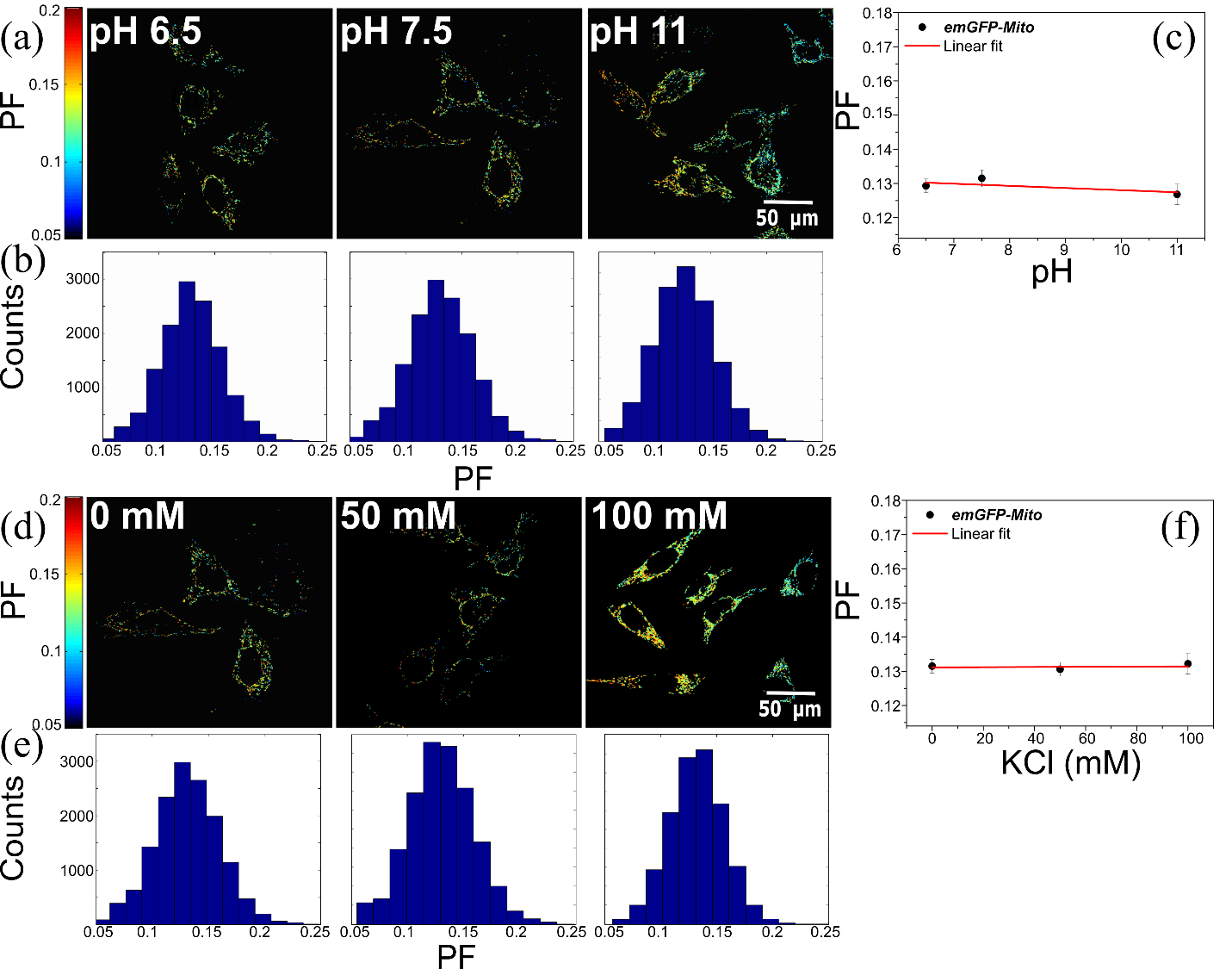


**Figure S7**. (a) pH dependence of PF parameter in emGFP-Mito at 23 ºC with (b) respective histograms and (c) mean value calculated from entire image. (d) Ionic strength dependence of PF parameter in emGFP-Mito with (e) representative histograms and (f) mean value.


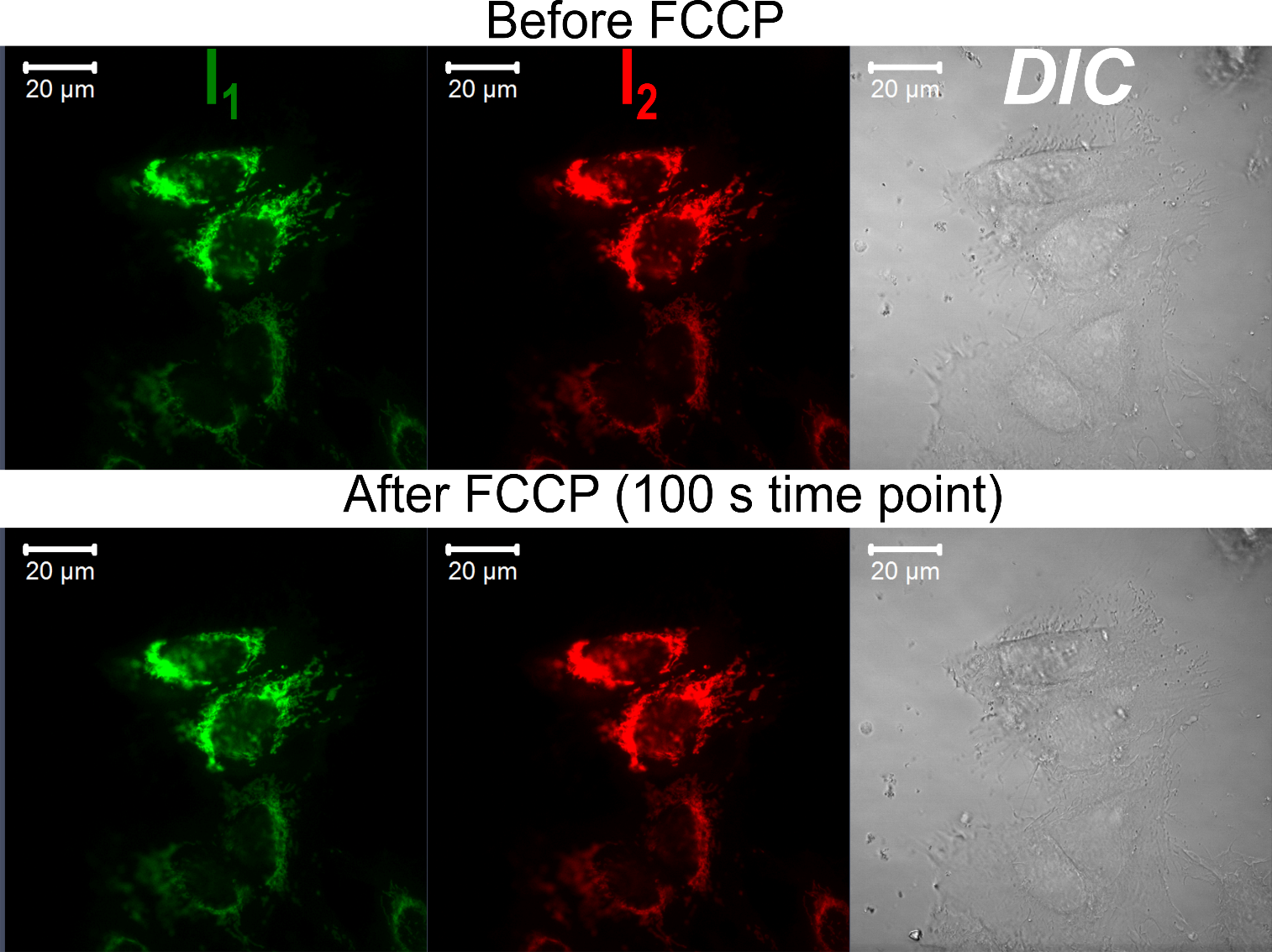


**Figure S8.** Multicolor confocal fluorescence intensity images of emGFP-Mito expressing HeLa cells recorded before and after 100s of FCCP treatment and simultaneously in the channel I_1_: 495 – 509 nm (left), I_2_: 510 – 600 nm (middle) and in differential interference contrast (DIC) mode (right).


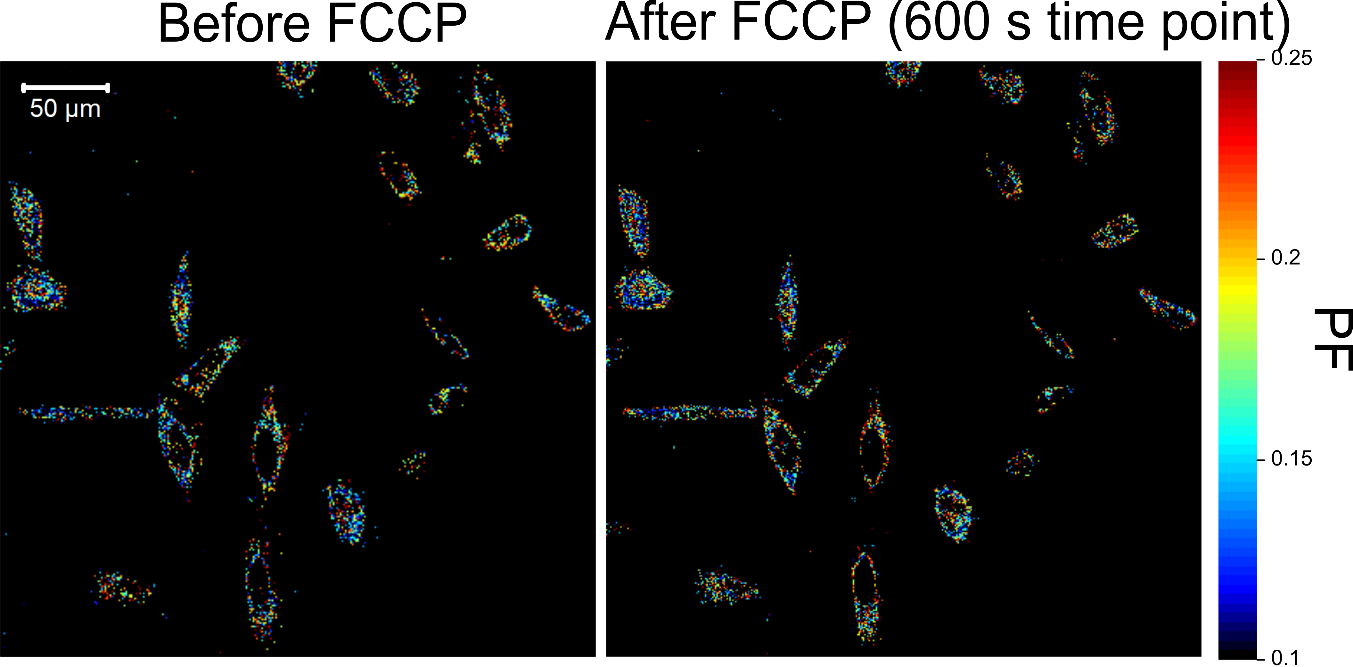


**Figure S9**. Peak fraction confocal fluorescence images of emGFP-Mito expressing HeLa cells recorded before and 600 s after FCCP treatment using a 40 x microscope objective. The peak fraction parameter equals to PF= I_2_-I_1_/I_total_.


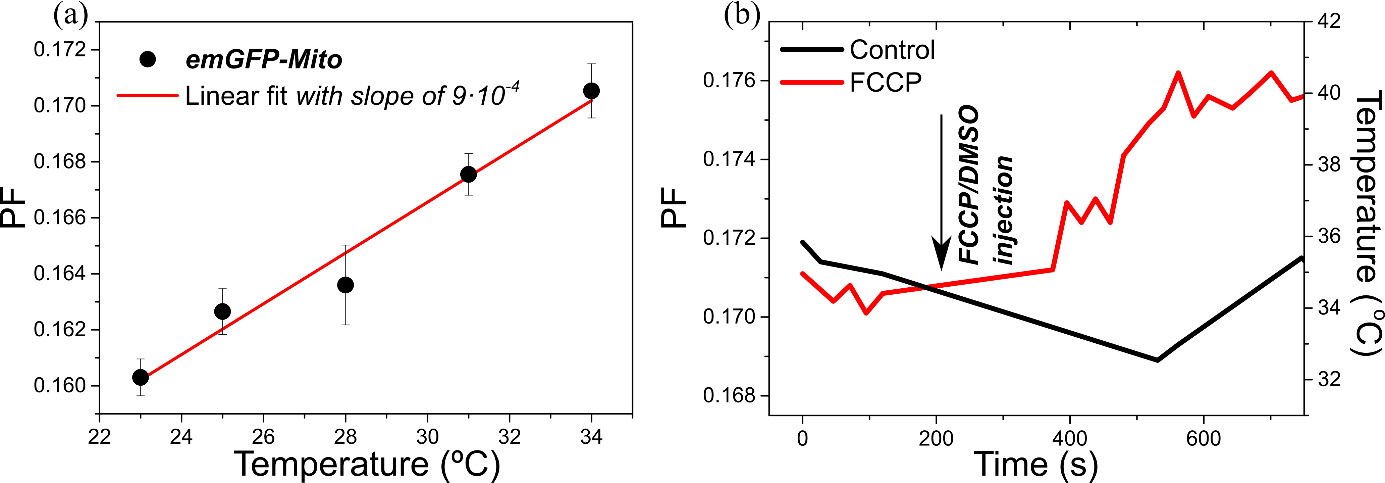


**Figure S10**. Fluorescence peak fraction nanothermometry using emGFP-Mito during FCCP treatment: (a) Calibration curve for the PF parameter recorded with the 40x microscope objective; (b) Time-dependent evolution of the peak fraction parameter: PF= I_2_-I_1_/I_total_ during FCCP treatment. The PF was determined from a series of images (see Fig. S5) averaging over the entire image. On the right y axis the resulting intracellular temperature is given determined using the calibration curve in (a).
